# Bibliographical

**Published:** 1859-08

**Authors:** 


### BIBLIOGRAPHICAL.

We find upon our table "Elements of Medicine," by Samuel Henry Dickson, M. D., Professor of the Practice of Physic in Jefferson Medical College, formerly Prof. of the Institutes and Practice of Physic in the Medical College of the State of South Carolina, and of the Theory and Practice of Medicine in the University of New York. This is a second edition of Prof. Dickson's "Compendious View of Pathology and Therapeutics, or the History and Treatment of Diseases," first issued in 1855, and which met at the time with general and high commendation from the medical press and the profession. The author in his preface to the second edition says, "I have endeavored to supply the defects pointed out, to remove redundances indicated, and to correct such errors as were commented on by my friendly critics or discovered on my own part after the most careful revision." And while we have not had an opportunity to examine this new edition critically, yet from the high character of the author, we do not doubt that this is as it professes to be, a greatly improved edition. It has been received from the publishers, Messrs. Blanchard & Lea, through the courtesy of J. J. Richards & Co., Booksellers of this city.

We have also received from the same publishers, through the hands of the Messrs. Richards, "Woman: her Diseases and Remedies;" a series of letters to his class, by Chas. D. Meigs, M. D., Prof. of Midwifery and the Diseases of Women and Children in the Jefferson Medical College, of Philadelphia, &c. This is the fourth edition, enlarged and revised, of that most unique and interesting volume, first published in 1850. It is only necessary to say that it is not only a deeply interesting book, but one which conveys an immense amount of practical knowledge in a most entertaining style.

We also announce from the same source a new American edition from the seventh revised and corrected London Edition of the Manual of Elementary Chemistry, Theoretical and Practical, by George Fowne, F. R. S., late Professor of Practical Chemistry in University College, London, edited by Robert Bridges, M. D., Prof. of Chemistry in the Philadelphia College of Pharmacy.

This work was first published in 1847, and soon obtained and has continued to enjoy the highest favor as a guide for the student in acquiring a knowledge of chemical science.

We also announce Anatamy Descriptive and Surgical, by Henry Gray, F. R. S., Lecturer on Anatomy at St. George's Hospital. The Drawings by H. V. Carter, M. D., late Demonstrator of Anatomy at St. George's Hospital.—The Dissections, jointly by the Author, and Dr. Carter; with three hundred and sixty three engravings on wood.—Philadelphia, Blanchard & Lea, 1857. The novel plan of the work, and the high character which the English Edition has acquired, with the improvements in the American Edition, commend this work to the confidence of both teachers and students.

"This work is intended to furnish the Student and Practitioner with an accurate view of the Anatomy of the Human Body, and more especially the application of this science to Practical Surgery.

One of the chief objects of the Author has been, to induce the Student to apply his anatomical knowledge to the more practical points in Surgery, by introducing, in small type, under each subdivision of the work,such observations as show the necessity of an accurate knowledge of the part under examination.

*Osteology.* Much time and care have been devoted to this part of the work, the basis of anatomical knowledge. It contains a concise description of the anatomy of the bones, illustrated by numerous accurately lettered engravings, showing the various markings and processes on each bone. The attachments of each muscle are shown in dotted lines (after the plan recently adopted by Mr. Holden), copied from recent dissections. The articulations of each bone are shown on a new plan; and a method has been adopted, by which the hitherto complicated account of the development of the bones is made more simple.

*The articulations.* In this section, the various structures forming the joints are described; a classification of the joints is given, and the anatomy of each carefully described: abundantly illustrated by engravings, all of which are taken from, or corrected, recent dissections.

*The Muscles and Fasciæ.* In this section, the muscles are described in groups, as in ordinary anatomical works. A series of illustrations, showing the lines of incision necessary in the dissection of the muscles in each region, are introduced, and the muscles are shown in fifty-seven engravings. The Surgical Anatomy of muscles in connection with fractures, and of the tendons or muscles divided in operations, is also described and illustrated.

*The Arteries.* The course, relations, and Surgical Anatomy of each artery are described in this section, together with the anatomy of the regions containing the arteries more especially involved in surgical operations. This part of the work is illustrated by twenty-nine engravings.

*The Veins* are described as in ordinary anatomical works; and illustrated by a series of engravings, showing those in each region.—The veins of the spine are described and illustrated from the well-known work of Breechet.

*The Lymphatics* are described, and figured in a series of illustration copied from the elaborate work of Mascagni.

*Nervous System and Organs of Sense.* A concise and accurate description of this important part of anatomy has been given, illustrated by sixty-three engravings showing the spinal cord and its membranes; the anatomy of the brain, in a series of sectional views; the origin, course, and distribution of the cranial, spinal, and sympathetic nerves; and the anatomy of the organs of sense.

*The Organs of Digestion, of Circulation, of Voice and Respiration, and the Urinary and Generative Organs.* A detailed description of this essential part of anatomy has been given, illustrated by fifty-three large and accurately lettered engravings.

*Regional Anatomy.* The anatomy of the perinæum, of the ischio-rectal region, and of femoral and inguinal herniæ, is described at the end of the work; the region of the neck, the axilla, the bend of the Scarpa's triangle, and the popliteal space, in the section on the arteries; the laryngo-tracheal region, with the anatomy of the trachea and larynx. These regions are illustrated by many engravings.

*Microscopical Anatomy.* A brief account of the microscopical anatomy of some of the tissues, and of the various organs, has also been introduced."

And finally we desire to direct the special attention of our readers to "Urinary Deposits," their Diagnosis, Pathology and Therapeutical Indications, by Golding Bird, M. D., F. R. S. Edited by Edmond Loyd Burkett, M. D., &c., &c., &c. A new American from the fifth London Edition, with eighty Illustrations on wood; Philadelphia, Blanchard & Lea. Through J. J. Richards & Co., Atlanta, Ga. To those who desire to avail themselves of the immense and inestimable contributions which have been made of late years to Urinary Pathology, and their important bearings on the practical part of the science of medicine, we can most confidently recommend this work, as of incalculable value. A mere statement of its contents, in connection with a knowledge of the source from which it proceeds, should be enough to secure it a place in the library of every medical man to which our Journal goes. The work contains a preliminary sketch of the Chemistry of the Urine—Physiological Origin and Physical Properties of the Urine—Chemical Physiology of the Urine—Chemical Pathology of Uric Acid—Chemical Pathology of Uric Oxide—Chemical Pathology of Purpurine—Chemical Pathology of Cystine—Chemical Pathology of Hippuric Acid—Chemical Pathology of Oxalate of Lime—Chemical Pathology of the Earthy Salts—Chemical Pathology of Abnormal Pigments—Pathology of Noncrystalline Organic Deposites—Therapeutic action of Remedies Influencing the Functions of the Kidneys; and Blood Depuration by the Kidneys, as a Remedy in Disease. In the last two chapters, especially, there are a number of practical observations and conclusions bearing upon the establishment of a more rational and philosophical system of Therapeutics, in connection with the removal of disease by the use of medicinal agents, which influence the action of the kidneys, which are of the highest value.—This is a book, for which, indeed, we feel that too much can not be said.

